# Supplementary material for: Epicardium-derived cells organize through tight junctions to replenish cardiac muscle in salamanders
Source: Nat Cell Biol. 2022 May 12;24(5):645–58. doi: 10.1038/s41556-022-00902-2 (PMC9106584; doi:10.1038/s41556-022-00902-2)
Supplement: Supplementary file 1 — Reporting Summary [file 41556_2022_902_MOESM1_ESM.pdf]

nature portfolio

Corresponding author(s): Elif Eroglu, András Simon, Kenneth R. Chien

Last updated by author(s): March 5th, 2022

Reporting Summary

Nature Portfolio wishes to improve the reproducibility of the work that we publish. This form provides structure for consistency and transparency in reporting. For further information on Nature Portfolio policies, see our Editorial Policies and the Editorial Policy Checklist.

Statistics

For all statistical analyses, confirm that the following items are present in the figure legend, table legend, main text, or Methods section.

n/a	Confirmed	
☐	☒	The exact sample size (*n*) for each experimental group/condition, given as a discrete number and unit of measurement
☐	☒	A statement on whether measurements were taken from distinct samples or whether the same sample was measured repeatedly
☐	☒	The statistical test(s) used AND whether they are one- or two-sided<br>*Only common tests should be described solely by name; describe more complex techniques in the Methods section.*
☒	☐	A description of all covariates tested
☐	☒	A description of any assumptions or corrections, such as tests of normality and adjustment for multiple comparisons
☐	☒	A full description of the statistical parameters including central tendency (e.g. means) or other basic estimates (e.g. regression coefficient) AND variation (e.g. standard deviation) or associated estimates of uncertainty (e.g. confidence intervals)
☐	☒	For null hypothesis testing, the test statistic (e.g. *F*, *t*, *r*) with confidence intervals, effect sizes, degrees of freedom and *P* value noted<br>*Give P values as exact values whenever suitable.*
☒	☐	For Bayesian analysis, information on the choice of priors and Markov chain Monte Carlo settings
☒	☐	For hierarchical and complex designs, identification of the appropriate level for tests and full reporting of outcomes
☒	☐	Estimates of effect sizes (e.g. Cohen's *d*, Pearson's *r*), indicating how they were calculated

Our web collection on statistics for biologists contains articles on many of the points above.

Software and code

Policy information about availability of computer code

Data collection	Images were acquired using Zen Blue (Carl Zeiss, v3.1). Echocardiography data was collected using the Vevo Lab 3.2.0. FACS data was collected using FACSDiva (BD Biosciences, v8.0.3).
Data analysis	-Sc-RNA-seq reads were mapped using STAR (v.2.5.3a).<br>-Single cell RNA sequencing analysis was performed using Seurat package version 4.0.1. All functions and parameters were described in detail in the Methods and Supplementary Information. All scripts are available upon request.<br>-Top 300 marker genes with the highest log fold change and a p-value < 0.05 were subjected to overrepresentation analysis using the Protein ANalysis THrough Evolutionary Relationships (PANTHER, Version 15) tool.<br>-Multiple sequence alignment of CLDN6 was performed with T-Coffee in default setting using the EMBL-EBI.<br>-Phylogenic tree was generated using the ClusterW2 package in the EMBL-EBI API. The alignment and tree were visualized using Jalview and TreeDyn.<br>-Monocle 3 (version 0.2.3.0) was used for single-cell trajectories and pseudo-time analysis, based on the UMAP generated in the Seurat analysis.<br>-ggplot2 R package (version 3.3.5) was used to generate scatter plots, bar plots, dot plots, violin plots and other data representation graphs.<br>-Statistical tests were performed using Graphpad Prism 9.1.2.<br>-Echocardiography data was analyzed using Vevo Lab 3.2.0.<br>-Images were analyzed using Zen Blue (Carl Zeiss, v3.1) and CaseViewer (v2.2.1).<br>-FACS data was analyzed using FACSDiva (BD Biosciences, v8.0.3) and FlowJo (v10.7.1).<br>-HALO (V3.0.311.267) software was used to quantify the RNAscope data.<br>-Image J (Fiji, version 2.0.0-rc-69/1.52p) was used to quantify the amount of Cre signal in cell nuclei.

For manuscripts utilizing custom algorithms or software that are central to the research but not yet described in published literature, software must be made available to editors and reviewers. We strongly encourage code deposition in a community repository (e.g. GitHub). See the Nature Portfolio guidelines for submitting code & software for further information.

Data

Policy information about availability of data

All manuscripts must include a data availability statement. This statement should provide the following information, where applicable:

- Accession codes, unique identifiers, or web links for publicly available datasets
- A description of any restrictions on data availability
- For clinical datasets or third party data, please ensure that the statement adheres to our policy

Sequencing data that support the findings of this study have been deposited at the Gene Expression Omnibus (GEO) under the accession code GSE180914. http:// PANTHER GO-SLIM database and REACTOME pathway database are publicly available (www.pantherdb.org/panther/goSlim.jsp, https://reactome.org/). Source data are provided with this study. All other data supporting the findings of this study are available from the corresponding authors on reasonable request.

Field-specific reporting

Please select the one below that is the best fit for your research. If you are not sure, read the appropriate sections before making your selection.

☒ Life sciences ☐ Behavioural & social sciences ☐ Ecological, evolutionary & environmental sciences

For a reference copy of the document with all sections, see nature.com/documents/nr-reporting-summary-flat.pdf

Life sciences study design

All studies must disclose on these points even when the disclosure is negative.

Sample size	No statistical methods were used to predetermine sample sizes. For both experiments involving animals and/or cell lines sample sizes were empirically estimated on the basis of pilot experiments and previously performed experiments with similar setup to provide sufficient sample sizes for statistical analysis. For experiments where animal to animal variation was high we typically employed n > 8 animals. For experiments where animal to animal variation was low we typically employed n < 8 animals.
Data exclusions	For the analysis of the scRNA-seq data: Genes expressed in less than three cells and cells with less than 200 expressed genes were omitted. Cells with total read counts lower than 5000 were also discarded.<br>For in vivo experiments no data were excluded, but sometimes an animal died during the course of the experiment, presumably due to the side effects of toxin treatment. In those instances data obtained in the interim analyses were included even if it was not possible to perform an end-point analysis.
Replication	The experimental results were obtained from at least three-independent experiments. All attempts were successful.
Randomization	For the studies relating to the CPE toxin and its variants: all animals were pre-screened for heart function via echocardiography to exclude any inherent functional defects. Before the animals were distributed into treatment groups, echocardiography was performed again to measure the injury sizes. If the injury sizes were too large (larger than 28%) or too small (smaller than 17%) the animals were excluded. Animals with similar sized injuries were distributed into the treatment groups randomly. For experiments other than those involving animals, samples were randomly allocated to experimental groups.
Blinding	All experiments relating to the CPE and its variants were performed by two investigators. Echocardiography data was collected and analyzed blindly by the investigators.

Reporting for specific materials, systems and methods

We require information from authors about some types of materials, experimental systems and methods used in many studies. Here, indicate whether each material, system or method listed is relevant to your study. If you are not sure if a list item applies to your research, read the appropriate section before selecting a response.

Materials & experimental systems

n/a	Involved in the study	
☐	☒	Antibodies
☐	☒	Eukaryotic cell lines
☒	☐	Palaeontology and archaeology
☐	☒	Animals and other organisms
☒	☐	Human research participants
☒	☐	Clinical data
☒	☐	Dual use research of concern

Methods

n/a	Involved in the study	
☒	☐	ChIP-seq
☐	☒	Flow cytometry
☒	☐	MRI-based neuroimaging

Antibodies

Antibodies used

-guinea pig anti-Cldn6 (1:500, custom made against the peptides TASQPRSDYPSKNYV and CPKKDDHYSARYTATA)
-rabbit anti-Cldn6 (1:50, 107059, abcam)
-mouse anti-CLDN6 antibody (1:30, MA5-24076, clone 342927, Thermo Fisher)
-mouse anti-MYH-1 (1:200, MF-20 concentrate, Myeloma Strain: P3U-1, DSHB)
- chicken anti-Vimentin (1:200, AB5733, Millipore)
- rabbit anti-α-smooth muscle actin (1:100, ab5694, Abcam)
-rabbit anti-Cre recombinase (1:500, ab216262, abcam)
-rabbit anti-cytokeratin (1:250, 9377, abcam)
-rabbit anti-GFP (1:500, A-6455, Life Technologies)
-chicken anti-GFP (1:1000, ab13970, abcam)
-mouse anti-Cre recombinase (1:500, C7920, clone 2Q2151, US biological)
-rabbit anti-RFP (1:200, 600-401-379, Rockland)
-rat anti-RFP (1:200, M11217, clone 16D7, Life Technologies)
-mouse anti-α-actinin (1:800, A7811, clone EA-53, Sigma)
-Alexa-Fluor 488 Phalloidin (1:500, A12379, Thermo Fisher)
-rabbit isotype control (1:100, abcam, ab171870)
-Isolectin GS-IB4, Alexa Fluor 488 or Alexa Fluor 647 Conjugate (1:200, I21411 or I32450 Thermo Fisher)
-Highly cross-adsorbed Alexa Fluor conjugated secondary antibodies (1:1000, raised in goats, Thermo Fisher) were used. Specifically, anti-chicken 488 (A11039, lot no. 2079383), anti-chicken 647 (A21449, lot no. 1698677), anti-guinea pig 555 (A21435, lot no. 1711692), anti-mouse IgG1 488 (A21121, lot no. 2983196), anti-mouse IgG1 647 (A21240, lot no. 2092265), anti-mouse IgG2b 647 (A21241, lot no.2056280), anti-mouse IgG2b 488 (A21141, lot no. 2228625), anti-mouse IgG2b 568 (A21144, lot no. 2349089), anti-mouse IgG2b 647 (A21242, lot no. 2155295), anti-rabbit 488 (A11034, lot no. 2069632), anti-rabbit 568 (A11011, lot no. 2277758), anti-rabbit 647 (A21245, lot no. 2018272), anti-rat 568 (A11077, lot no. 1692966)

Validation

All antibodies are commercially available and validated by the manufacturer except for guinea pig anti-Claudin 6 (Chien Lab). Protein sequence conservation has been considered while choosing antibodies to be used on Pleurodeles waltl tissues. The subcellular localization of all the proteins analyzed in this study has been previously reported. This was used to validate the specificity of the antibody.

Guinea pig and rabbit anti-Cldn6 antibodies were validated via western blot on HEK293T cell extracts obtained from cells transfected with wild type Pleurodeles waltl Cldn6 or a deletion mutant.

Anti-MYH-1 (MF-20 concentrate, DSHB) has been validated on salamander tissue in Mercer et al., Developmental Biology, 2013.
Anti-Cre recombinase (C7920, US biological) has been validated on Pleurodeles tissue in Joven et al., Development, 2018.

-rabbit anti-Cldn6 (https://www.citeab.com/antibodies/723509-ab107059-anti-claudin-6-antibody)
-mouse anti-CLDN6 antibody (https://www.thermofisher.com/antibody/product/Claudin-6-Antibody-clone-342927-Monoclonal/MA5-24076)
-mouse anti-MYH-1 (MF-20 concentrate) (https://dshb.biology.uiowa.edu/MF-20)
-chicken anti-Vimentin (https://www.merckmillipore.com/SE/en/product/Anti-Vimentin-Antibody,MM_NF-AB5733)
- rabbit anti-α-smooth muscle actin (https://www.abcam.com/alpha-smooth-muscle-actin-antibody-ab5694.html)
-rabbit anti-Cre recombinase (https://www.abcam.com/cre-recombinase-antibody-ab216262.html)
-rabbit anti-cytokeratin (https://www.abcam.com/wide-spectrum-cytokeratin-antibody-ab9377.html)
-rabbit anti-GFP (https://www.thermofisher.com/antibody/product/GFP-Antibody-Polyclonal/A-6455)
-chicken anti-GFP (https://www.abcam.com/gfp-antibody-ab13970.html)
-mouse anti-Cre recombinase (https://www.usbio.net/antibodies/C7920/Cre-Recombinase)
-rabbit anti-RFP (https://www.rockland.com/categories/primary-antibodies/rfp-antibody-pre-adsorbed-600-401-379/)
-rat anti-RFP (https://www.thermofisher.com/antibody/product/mCherry-Antibody-clone-16D7-Monoclonal/M11217)
-mouse anti-α-actinin (https://www.sigmaaldrich.com/SE/en/product/sigma/a7811)
-Alexa-Fluor 488 Phalloidin (https://www.thermofisher.com/order/catalog/product/A12379)
-rabbit isotype control (https://www.abcam.com/rabbit-igg-polyclonal-isotype-control-chip-grade-ab171870.html)
-Isolectin GS-IB4, Alexa Fluor 488 Conjugate (https://www.fishersci.com/shop/products/molecular-probes-alexa-fluor-isolectin-gs-ib-sub-4-sub-from-i-griffonia-simplicifolia-i-alexa-fluor-488-conjugate/I21411)
-Isolectin GS-IB4, Alexa Fluor 647 Conjugate (https://www.fishersci.com/shop/products/molecular-probes-alexa-fluor-isolectin-gs-ib-sub-4-sub-from-i-griffonia-simplicifolia-i-alexa-fluor-647-conjugate/I32450)

Eukaryotic cell lines

Policy information about cell lines

Cell line source(s)	HEK293T cells were obtained from the ATCC.
Authentication	The cell line was obtained from ATCC and was not revalidated.
Mycoplasma contamination	We perform routine mycoplasma checks in the lab but this cell line was not tested for mycoplasma contamination.
Commonly misidentified lines (See ICLAC register)	We have checked the ICLAC register and the cell line used in our studies are not on the list of misidentified cell lines.

Animals and other organisms

Policy information about studies involving animals; ARRIVE guidelines recommended for reporting animal research

Laboratory animals	Post-metamorphic (up to a year old) male/female wild type or transgenic Pleurodeles waltl were used for the salamander experiments. Animals had mixed genetic background.<br>Transgenic lines are as listed below:<br>tgTol2(CAG:loxP-GFP-loxP-Cherry)Simon<br>tgTol2(CAG:loxP-Cherry-loxP-H2B::YFP)Simon<br>tgTol2(CAG:Nucbow)Simon
Wild animals	The study did not involve wild animals.
Field-collected samples	The study did not involve samples collected from the field.
Ethics oversight	All the procedures related to animal handling, care and the treatment in this study were performed according to the guidelines approved by the Jordbruksverket/Sweden under the ethical permit numbers 18190-18 and 5723-2019.

Note that full information on the approval of the study protocol must also be provided in the manuscript.

Flow Cytometry

Plots

Confirm that:

☒ The axis labels state the marker and fluorochrome used (e.g. CD4-FITC).

☒ The axis scales are clearly visible. Include numbers along axes only for bottom left plot of group (a 'group' is an analysis of identical markers).

☒ All plots are contour plots with outliers or pseudocolor plots.

☒ A numerical value for number of cells or percentage (with statistics) is provided.

Methodology

Sample preparation	To perform ventricle dissociations, hearts were collected and rinsed with ice cold amphibian HBSS (aHBSS, 70%) (Sigma, 55037C). Atria and outflow track were removed from the ventricle with the help of scissors. Using a sterile scalpel, ventricles were minced into smaller pieces and collected in aHBSS in an Eppendorf tube on ice. Tissue pieces were allowed to settle, rinsed once with aHBSS and incubated with 2 mg ml-1 Collagenase/Dispase (Sigma, 10269638001) in aHBSS for 2 hours at 27°C with frequent gentle tapping. After 2 hours, tissue pieces were carefully rinsed with aHBSS, resuspended in aPBS with 10% FBS and mechanically broken with the help of a pipette. Cells were passed through a 100 µm filter and spun down at 300g, 4°C for 5 minutes. The pellets were resuspended in 1 ml aPBS with 1% FBS. Cell viability and number was assessed via automated cell counter (TC20™ Bio-Rad). Cells were stained with Sytox™ Blue dead cell stain (1:1000, S34857, ThermoFisher), Calcein AM (1:250, C1430, ThermoFisher) and Vybrant™ DyeCycle™ Ruby (1:1000, V10273, ThermoFisher) according to the manufacturer’s protocols. Cells were sorted on a FACS Aria III system (BD Biosciences) using a 130 µm nozzle to accommodate larger salamander cell size.<br>In order to establish a milder dissociation protocol to capture the cells in the injury area at 7 dpci, apical regions of the hearts were removed with a scalpel and minced into smaller pieces that were collected in aHBSS in a flat-bottom borosilicate glass jar. Tissue pieces were rinsed once with cold aHBSS and incubated in digestion buffer containing 1.5 mg ml-1 bovine serum albumin (Sigma, A7906), 3 mg ml-1 glucose (Sigma, G6152), 2 mg ml-1 Collagenase/Dispase (Sigma, 10269638001) in aHBSS. Tubes were shaken in a 27°C waterbath for 30 minutes with 90 rpm shaking. After 30 minutes, solution containing tissue pieces and dissociated cells was gently pipetted up and down without disturbing sizeable tissue pieces and collected in a separate tube with FBS. Fresh digestion buffer was added to the remaining tissue pieces and the procedure was repeated after another 30 minutes.<br>To perform CLDN6 antibody staining on isolated cells, 5 ug CLDN6 antibody (abcam, 107059) or rabbit isotype control (abcam, ab171870) was conjugated to Dylight 650 using Dylight 650 Fast Conjugation Kit (abcam, 201803). Cells were incubated with the conjugated antibodies for an hour at 4°C and washed two times with FACS buffer to remove unbound antibodies. Sytox blue and vybrant orange staining was performed subsequently to stain for live, nucleated cells.
Instrument	FACS Aria III system (BD Biosciences), 130 um nozzle
Software	BD FACSDiva Software
Cell population abundance	Cells were collected on miscroscope slides to assess morphology and to make sure no doublets were present.

Gating strategy

Cells were first gated to exclude debris (FSC-A vs SSC-A plot), then gated to select for singlets (FSC-W vs FSC-A plot). To select for live, metabolically active and nucleated cells: Dead cells were excluded by gating for Sytox Blue negative cells (Sytox Blue vs FSC-A plot). Then metabolically active cells were determined to be Calcein-AM positive (Calcein-AM vs FSC-A plot) and lastly nucleated cells were selected to be Vybrant Ruby positive (Vybrant Ruby vs FSC-A).

Please refer to Extended Data Fig. 5a for a figure exemplifying the gating strategy described above.

☒ Tick this box to confirm that a figure exemplifying the gating strategy is provided in the Supplementary Information.
